# Caffeine and Rolipram Affect Smad Signalling and TGF-β1 Stimulated CTGF and Transgelin Expression in Lung Epithelial Cells

**DOI:** 10.1371/journal.pone.0097357

**Published:** 2014-05-14

**Authors:** Markus Fehrholz, Christian P. Speer, Steffen Kunzmann

**Affiliations:** University Children's Hospital, University of Würzburg, Würzburg, Germany; University of Tübingen, Germany

## Abstract

Caffeine administration is an important part of the therapeutic treatment of bronchopulmonary dysplasia (BPD) in preterm infants. However, caffeine mediated effects on airway remodelling are still undefined. The TGF-β/Smad signalling pathway is one of the key pathways involved in airway remodelling. Connective tissue growth factor (CTGF), a downstream mediator of TGF-β, and transgelin, a binding and stabilising protein of the cytoskeleton, are both regulated by TGF-β1 and play an important role in airway remodelling. Both have also been implicated in the pathogenesis of BPD. The aim of the present study was to clarify whether caffeine, an unspecific phosphodiesterase (PDE) inhibitor, and rolipram, a prototypical PDE-4 selective inhibitor, were both able to affect TGF-β1-induced Smad signalling and CTGF/transgelin expression in lung epithelial cells. Furthermore, the effect of transgelin knock-down on Smad signalling was studied. The pharmacological effect of caffeine and rolipram on Smad signalling was investigated by means of a luciferase assay via transfection of a TGF-β1-inducible reporter plasmid in A549 cells. The regulation of CTGF and transgelin expression by caffeine and rolipram were studied by promoter analysis, real-time PCR and Western blot. Endogenous transgelin expression was down-regulated by lentiviral transduction mediating transgelin-specific shRNA expression. The addition of caffeine and rolipram inhibited TGF-β1 induced reporter gene activity in a concentration-related manner. They also antagonized the TGF-β1 induced up-regulation of CTGF and transgelin on the promoter-, the mRNA-, and the protein-level. Functional analysis showed that transgelin silencing reduced TGF-β1 induced Smad-signalling and CTGF induction in lung epithelial cells. The present study highlights possible new molecular mechanisms of caffeine and rolipram including an inhibition of Smad signalling and of TGF-β1 regulated genes involved in airway remodelling. An understanding of these mechanisms might help to explain the protective effects of caffeine in prevention of BPD and suggests rolipram to be a potent replacement for caffeine.

## Introduction

Bronchopulmonary dysplasia (BPD) today is still one major challenge for preterm infants [1], [2]. BPD is characterized by a disruption of normal lung alveolarization and vascularisation, leading to fewer but larger alveoli and a simplification of the vascular structures [3]. BPD is clinically defined by requirement of supplemental oxygen at 36 weeks gestation [2]. The disease can be complicated by development of pulmonary hypertension (PH) [1]. The etiology of BPD is multifactorial, including extreme lung immaturity combined with lung injury implicating inflammatory and remodelling responses induced by mechanical ventilation, oxygen stress, and/or infection [3].

The TGF-β/Smad signalling pathway is one of the key pathways involved in lung development, airway inflammation, airway remodelling, and lung fibrosis [4], [5]. All of these processes contribute to the development of BPD [53],[56]. In BPD, increased expression of TGF-β and activation of Smad signalling could be described [7]–[11].

CTGF and transgelin, both downstream mediators of TGF-β and both regulated in a TGF-β/Smad3 dependent fashion [12], [13], contribute to TGF-β dependent airway/vascular remodelling processing in BPD [9], [11], [14]–[16]. Like for CTGF [17], in pulmonary fibrosis and pulmonary hypertension an increased transgelin expression could also be described [18]–[20]. While a direct influence of CTGF on Smad signalling was shown [21], an impairment of Smad signalling by transgelin is only hypothesized [22].

The incidence of bronchopulmonary dysplasia (BPD) can be reduced by the application of caffeine [23]. The molecular basis of the protective actions of caffeine and its metabolites (paraxanthine, theobromine, and theophylline) in airway remodelling processes of the neonatal immature lung is not well defined. In addition, the different molecular actions mediated by caffeine are highly dose dependent [24]. At high plasma concentrations, caffeine, like theophylline, acts as a non-selective phosphodiesterase (PDE) inhibitor, thereby leading to higher levels of the intracellular second messenger cyclic adenosine monophosphate (cAMP) [24]. cAMP was one of the first identified second messengers transmitting signals via G-protein-coupled receptors and protein kinase A (PKA) from the cell surface to the nucleus [25]. cAMP suppresses the activation of inflammatory cells like mast cells, eosinophils, neutrophils, monocytes, and lymphocytes. It also mediates a relaxation of airway smooth muscle through activation of PKA and PKB, thereby leading to bronchodilation [25]. The role of cAMP in airway remodelling processes, however, is less well defined.

Currently, there are at least eleven genetically different PDE iso-enzymes known [26]. Among the PDE iso-enzymes, PDE-4 is highly specific for cAMP and is its major metabolizing enzyme in the airways [26]. In addition, PDE-4 is also the dominant iso-enzyme in inflammatory cells, which underlines that specific PDE-4 inhibitors like rolipram, beside their actions as bronchodilators, would be useful as an anti-inflammatory treatment in different inflammatory pulmonary diseases. These might include asthma, chronic obstructive pulmonary disease (COPD), or BPD [24], [27]–[31]. The potential benefit of an unselective PDE inhibitor like caffeine or theophylline and a more specific PDE4-inhibitor like rolipram in diseases associated with abnormal tissue remodelling like BPD is insufficiently characterized.

In this context, the current study was designed to address the question if there exist possible effects of caffeine and rolipram on the Smad signalling pathway and on expression levels of TGF-β family members (CTGF and transgelin) involved in airway remodelling processes in lung epithelial cells.

## Methods

### Reagents

Recombinant TGF-β1 was obtained from R&D Systems (Abingdon, UK). Caffeine, rolipram, and dibutyryl-cAMP (db-cAMP) were purchased from Sigma-Aldrich (St. Louis, CA).

### Cells

A549 cells, a human lung carcinoma cell line with characteristics of human alveolar basal epithelial cells, were purchased from ATCC (LGC Standards, Teddington, UK) [32]. A549 cells were cultured in RPMI 1694 and DMEM (Gibco, Life Technologies, Carlsbad, CA) with additional 5% fetal bovine serum, 100 U/mL penicillin, and 100 mg/mL streptomycin (Gibco). Experiments with TGF-β1 were performed in serum-free medium. Incubation was carried out at 37°C in a humidified atmosphere with 5% CO_2_.

### Cell viability assay

A549 cell viability after exposure to TGF-β1 (10 ng/ml), caffeine (10 mM), rolipram (100 µM), and db-cAMP (10 mM) was evaluated after 1–3 days using methylthiazolyldiphenyl-tetrazolium bromide (MTT). Cells were seeded in six well plates (Greiner) and treated as described. After washing with Dulbecco's Phosphate Buffered Saline (DPBS; Sigma-Aldrich), 1 mL DPBS containing 1.2 mM MTT was applied to the cells and the plates were incubated at 37°C for 30 min. The MTT-medium was then removed, 500 µL isopropanol was added to the wells, and plates were gently rocked for 5 min at RT to dissolve purple formazan from vital cells. Optical density was measured in triplicates in 96 well plates (Greiner) with an MR 5000 microplate reader (Dynatech, Santa Monica, CA) at 550 nm. Untreated cells were considered 100% vital and used as reference.

### Transfection and promoter assays

The human transgelin (TAGLN) promoter sequence (GenBank ID EF153019.1) was cloned into the pGL3 Basic vector (Promega, Fitchburg, WI) between BglII and HindIII sites by amplification from A549 genomic DNA using primers BglIITAGLNprmtFwd 5′-CACCAGATCTGTCCAGGGATCCCACTGTTAGTC-3′ and HindIIITAGLNprmtRev 5′-CCTAAAGCTTAGGCTTCCTCAGGGCTCGC-3′, respectively. Cloning of the (CAGA)_12_-and p3TP-luciferase plasmid were described before [33], [34]. The CAGA elements were originally found in the promoter region of plasminogen activator inhibitor-1 and are known to be activated after binding of the Smad3/4 complexes and after TGF-β1 binding to the TGF-β receptor [33]. (CAGA)_12_-luc (2 µg), p3TP-luc (2 µg) or the transgelin-promotor vector (2 µg), and Renilla luciferase control reporter vector (phRL-TK; 5 ng) were transfected into A549 cells, seeded in 6-well plates, using 6 µg of linear polyethylenimine MW 25,000 (Polysciences Inc., Warrington, PA). Transfection medium (Optimem, Invitrogen) was changed to Optimem with additional 0.2% fetal bovine serum after 2 hours. Twenty-four hours after transfection, cells were treated with indicated molecules. Then 16 hours later, luciferase activity was measured by the dual luciferase assay system (Promega Biotech Inc., Madison, Wis) according to the manufacturer's instruction using a Berthold MiniLumat LB 9506 luminometer (Bad Wildbach, Germany). Firefly luciferase activity was normalized to the activity of Renilla luciferase under control of thymidine kinase promoter of phRL-TK. Results are given as relative increase compared to controls. All values were obtained from experiments carried out in triplicates and repeated at least three times. The error bars indicated standard error of the mean (SEM).

### RNA extraction and RT-PCR

For RNA extraction, 3×10^5^ A549 cells were seeded on six well plates (Greiner, Frickenhausen, Germany) and grown at 37°C. Cells were washed with DPBS and treated as indicated. After the appropriate time, cells were washed again and total RNA was isolated using NucleoSpin RNA II Kit (Macherey-Nagel, Dueren, Germany) according to the manufacturer's protocol. Total RNA was eluted in 60 µL nuclease-free H_2_O and stored at -80°C until reverse transcription. For RT-PCR, 1 µg of total RNA was reverse transcribed using High Capacity cDNA Reverse Transcription Kit (Applied Biosystems, Life Technologies, Carlsbad, CA) according to the manufacturer's instructions. Upon analysis, first strand cDNA was stored at −20°C.

### Quantitative real time RT-PCR (qPCR)

For detection of human CTGF, transgelin, and GAPDH mRNA, cDNA was analyzed using 12.5 µL iQ™ SYBR Green Supermix (Bio-Rad Laboratories, Hercules, CA), 0.5 µL deionized H_2_O, and 10 pmol of each forward and reverse primer, respectively. Primers for CTGF, transgelin, and GAPDH mRNA were hCTGFfwd 5′-ACCCAACTATGATTAGAGCC-3′, hCTGFrev 5′-TTGCCCTTCTTAATGTTCTC-3′, hTAGLNfwd 5′-CGAGAAGAAGTATGACGAGG-3′, hTAGLNrev 5′-CTTGCTCAGAATCACGCC-3′, hGAPDHfwd 5′-CAAAGTTGTCATGGATGACC-3′, and hGAPDHrev 5′-CCATGGAGAAGGCTGGGG-3′, respectively, and were designed based on genomic and mRNA sequences for CTGF (accession numbers NG_016131 and NM_001901.2), transgelin (accession numbers EF445034.1 and NM_001001522.1), and GAPDH (accession numbers NG_007073.2 and NM_002046.5) using PerlPrimer software version 1.1.20. Real-time PCRs were performed on an ABI Prism 7500 Sequence Detection System (*Taq*Man) as described [35]. Melt curve analyses were performed to verify single PCR products. Results were normalized to GAPDH and mean fold changes were calculated by the ΔΔC_T_ method [36].

### Western blot analysis

A549 cells were rinsed with ice-cold tris-buffered saline (TBS) and incubated in 100 µl lysis buffer consisting of Cell lysis buffer (Cell Signaling Technologies, MA), Complete Mini protease inhibitor cocktail tablets and PhosStop phosphatase inhibitor cocktail tablets (Roche, Germany), and 0.1 mM PMSF (Merck KGaA, Germany) for 10 minutes on ice. The lysate was cleared by centrifugation at 30,000 g for 10 min, and the supernatant was used for Western immunoblotting analysis. Protein concentrations were determined for each sample using the Bradford assay (Bio-Rad, Richmond, CA). Equal amounts of cellular protein were loaded and separated by SDS-PAGE on 10% to 12% Bis-Tris gels and electrophoretically transferred to polyvinylidene difluoride or nitrocellulose blotting membranes (Amersham Pharmacia Biotech, Piscataway, NJ). Membranes were blocked in 5% BSA for 1 hour at room temperature and successively incubated with primary antibodies overnight at 4°C. Western blots were probed with primary antibodies to CTGF (ab6992; Abcam, Cambridge, UK), transgelin (sc-50446; Santa Cruz Biotechnology, Santa Cruz, CA), Smad2/3-P (rabbit anti-Smad2/3-P was a gift from Dr. C.-H. Heldin (Ludwig Institute for Cancer Research, Uppsala, Sweden)) and β-actin (926–42212; LI-COR Inc., Lincoln, NE), followed by staining with corresponding IRDye secondary antibodies (LI-COR Inc.) for 1 h at room temperature. Specific protein bands were visualized using an Odyssey Infrared Imaging System (LI-COR Inc.). Accumulated signals were quantifieded using Odyssey Software v2.1 (LI-COR Inc.).

### Transgelin Gene Silencing by Lentiviral Transduction

For expression of transgelin-specific shRNA, the lentiviral system as described by Zinke et al. was used [37]. The transgelin specific siRNA sequence 5′-GGCTCTGTCACTGAGCAAT-3′ and the inactive control siRNA (scrmbl) sequence 5′-CCAGAGCTATCTCAGATAG-3′, respectively, embeded in the cellular human miR30 pre-micro-RNA backbone, were introduced into a modified, shRNA-expressing F6GW vector using HpaI and XhoI sites. F6GW was modified to contain a puromycin N-acetyl-transferase sequence instead of those for EGFP using BamHI and EcoRI sites. The correct sequence of all constructs was verified by sequencing. Vesicular stomatitis virus envelope protein G (VSV-G)-pseudotyped lentiviral particles were produced by transfection of 293T cells with plasmids F6GW-shRNATAGLN or F6GW-shRNAscrmbl, respectively, pMDLg, pRSV-Rev, and pMD.G as described [37]. Sterile-filtered particles were further concentrated using 100 kDa Amicon Ultra-15 filters (Merck Millipore, Billerica, MA). After transduction with shRNA-expressing lentiviral vectors, A549 cells were expanded in culture medium containing 2 µg/mL puromycin. No differences on cell morphology and proliferation were observed between transduced cells.

### Data analysis

All results shown are representative of three separate experiments. Results are given as means ±SEM. Data were analyzed by the Mann-Whitney-Wilcoxon test. A p-value <0.05 was considered significant. All statistical analyses were performed using the statistical software GraphPad Prism 5.0.

## Results

### Effect of caffeine, rolipram, and db-cAMP on TGF-β1 induced Smad-signalling in lung epithelial cells

**Figure 1:**
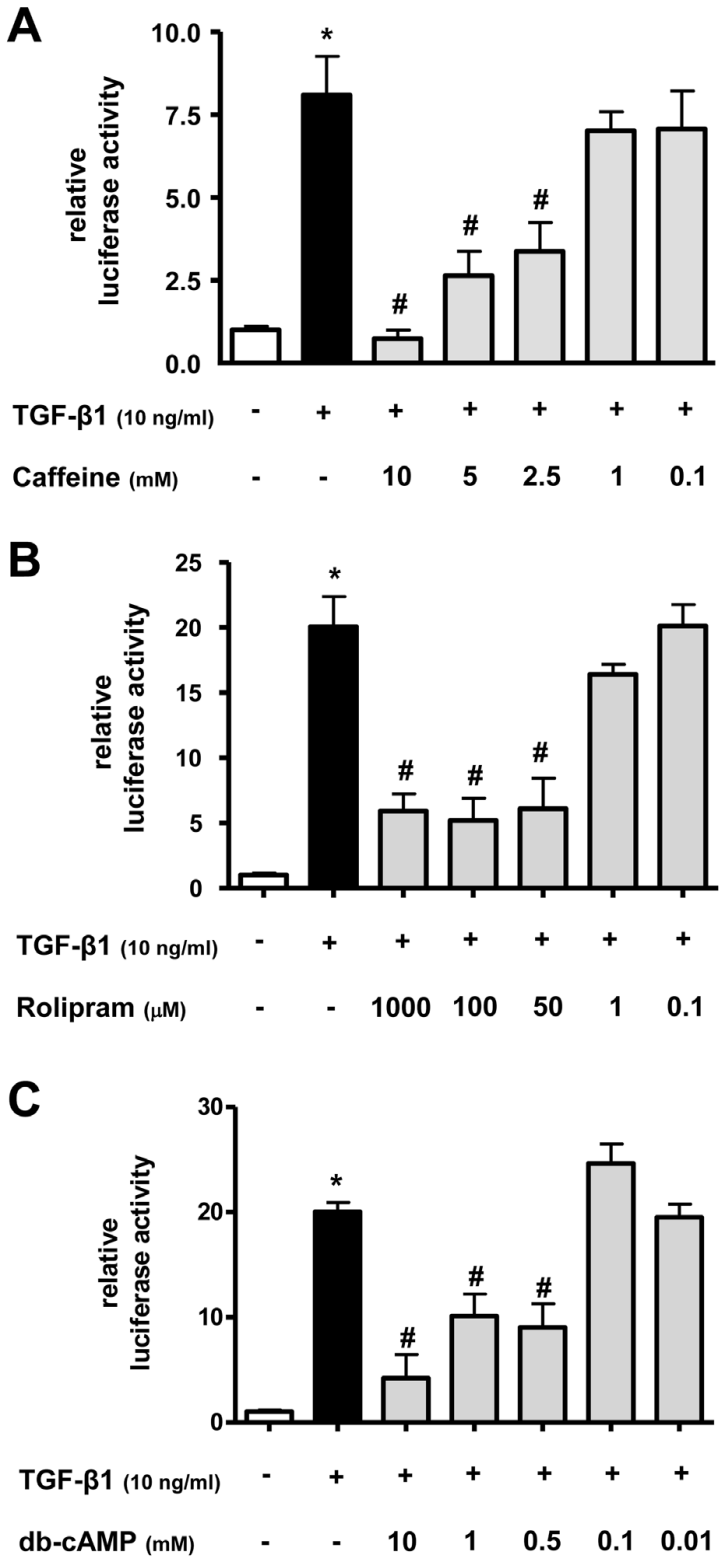
Caffeine, rolipram, and db-cAMP inhibit TGF-β1 induced Smad-signalling in lung epithelial cells. The TGF-β1–sensitive (CAGA)_12_-luciferase reporter construct was transiently transfected into A549 cells. Cells were then treated with TGF-β1 (10 ng/ml) and/or different concentrations of caffeine (A), rolipram (B), or db-cAMP (C). Firefly luciferase activity was normalized to the activity of Renilla luciferase under control of the thymidine kinase promoter. Relative luciferase activity compared to controls is shown. * p<0.05 compared to control cells, # p<0.05 compared to TGF-β1 treated cells.

To investigate the possible effect of caffeine, rolipram, and the membrane-permeable cAMP analog dibutyryl-cyclic AMP (db-cAMP) on Smad-signalling in lung epithelial cells, a TGF-β1–sensitive (CAGA)_12_-luciferase construct was transfected into lung epithelial cells A549. As a positive control, TGF-β1 induced a significant increase of reporter gene activity compared to untreated lung epithelial cells A549 using the (CAGA)_12_-luciferase construct (Figure 1A–C) (p<0.05). Caffeine (Figure 1A), rolipram (Figure 1B), and db-cAMP (Figure 1C) inhibited the TGF-β1 induced reporter gene activity in a concentration-related manner. Caffeine at 10 mM was able to antagonize the effect of TGF-β1 on Smad activation completely (p<0.05) (Figure 1A). Rolipram, used in a concentration of 100 µM, reduced TGF-β1 induced Smad activity by 75±13% (p<0.05) (Figure 1B) and db-cAMP at 10 mM reduced Smad activity by 80±18% (p<0.05) (Figure 1C).

Taken together, these results showed that TGF-β1 is able to activate the Smad signalling pathway in A549 cells. In turn, the unspecific PDE inhibitor caffeine, the more specific PDE-4 inhibitor rolipram, and the cAMP analog db-cAMP were able to inhibit the effect of TGF-β1 on Smad signalling activation in lung epithelial cells.

### Effect of caffeine and rolipram on TGF-β1 induced CTGF expression in lung epithelial cells

**Figure 2:**
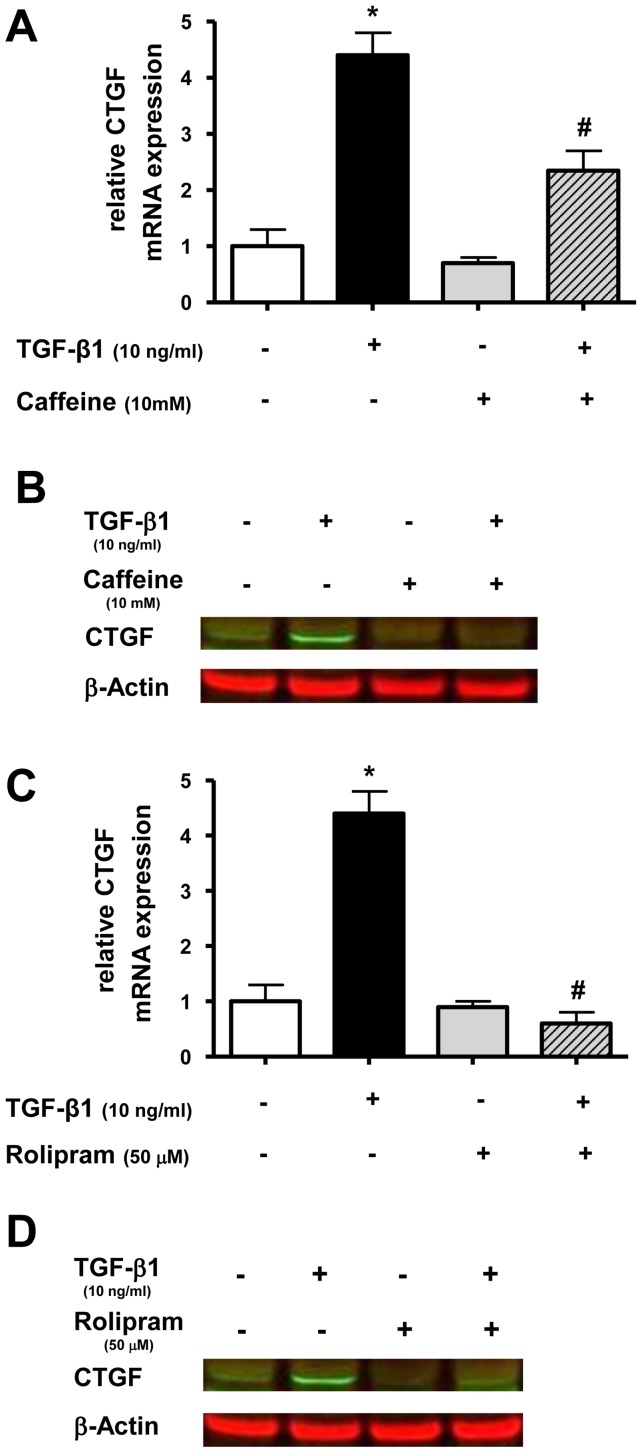
Caffeine and rolipram inhibit TGF-β1 induced CTGF expression in lung epithelial cells. A549 cells were incubated with TGF-β1 (10 ng/ml) and with or without caffeine (10 mM) or rolipram (50 µM). Real-time PCR of CTGF mRNA was performed after 12h (A+C) and Western blot analysis after 24 h (B+D). Relative mRNA levels of CTGF were calculated by normalizing signals to detected GAPDH mRNA. Differences compared to untreated cells were calculated (A+C). Means±SD of at least n = 3 independent experiments are shown. In B+D representative immunoblots of at least three independent experiments are shown. * p<0.05 compared to control cells, # p<0.05 compared to cells treated with TGF-β1.

As a next step, we wanted to study the effect of caffeine and rolipram on expression of the TGF-β1 regulated gene CTGF in lung epithelial cells. A549 cells were treated with TGF-β1 and with or without caffeine or rolipram. CTGF mRNA and protein expression was measured 12 or 24 hours later, respectively. TGF-β1 alone increased CTGF mRNA levels 4.4-fold compared to untreated cells (p<0.05) (Figure 2A+C). Caffeine and rolipram alone had no significant effect on CTGF mRNA expression (Figure 2A+C). TGF-β1-induced CTGF expression was reduced to 54±7% (p<0.05) by caffeine (Figure 2A) and completely by rolipram (p<0.05) (Figure 2C). The inhibitory effect of caffeine and rolipram on TGF-β1 mediated CTGF mRNA up-regulation was confirmed on the protein level by immunoblotting (Figure 2B+D).

These results demonstrated that TGF-β1 is able to enhance CTGF expression in A549 cells, which was diminished by caffeine and abolished by rolipram.

### Regulation of transgelin by TGF-β1 and caffeine in lung epithelial cells

Next we examined the effect of TGF-β1 and caffeine on transgelin expression in A549 cells.

**Figure 3:**
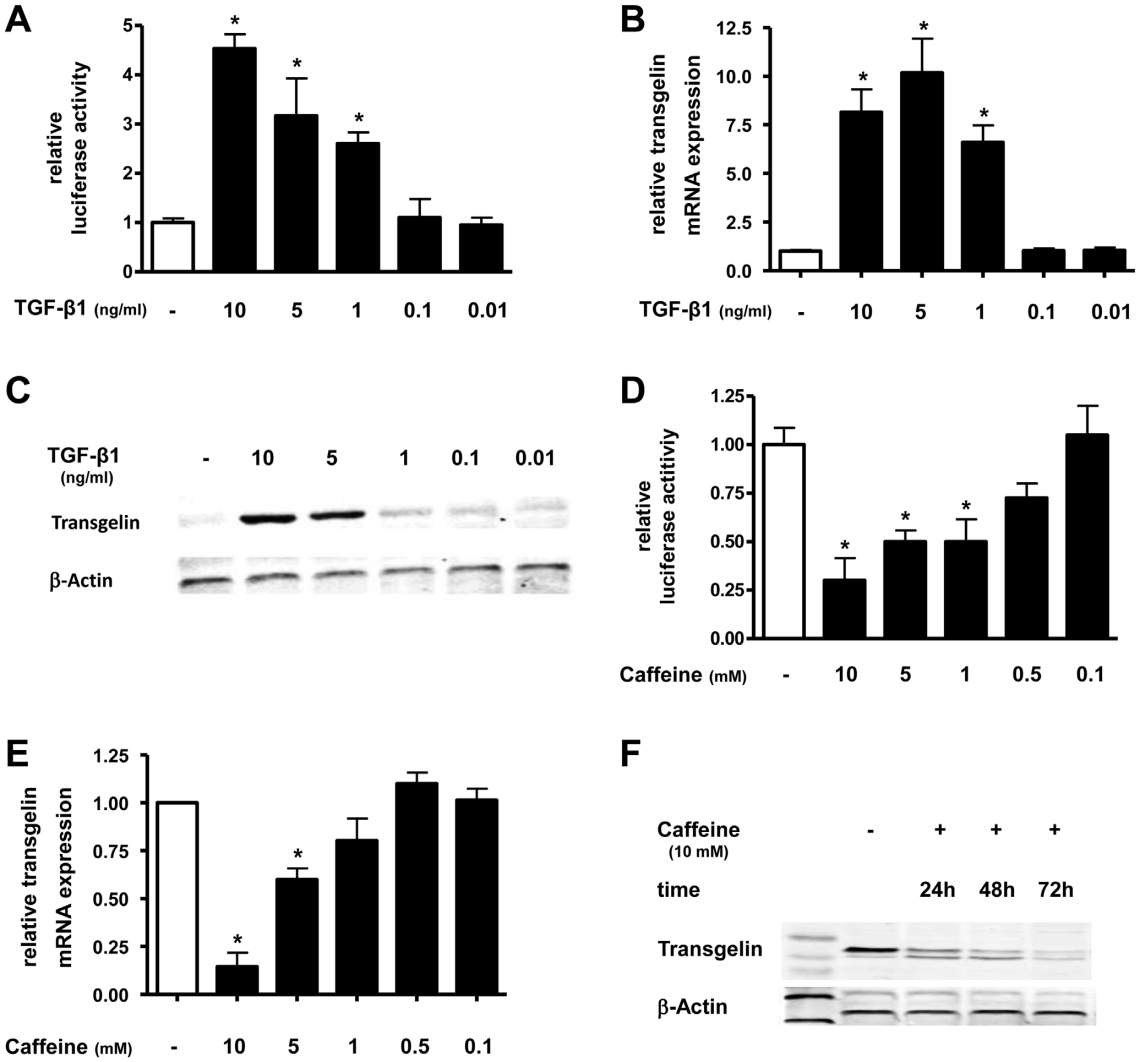
TGF-β1 increases and caffeine decreases transgelin expression in lung epithelial cells. A549 cells were incubated with TGF-β1 (A–C) or caffeine (D–F) in indicated concentrations and for various timepoints. Transgelin promoter (A+C), real-time PCR (B+E) and Western blot (C+F) analysis was performed. Means±SD of at least n = 3 independent experiments are shown. In C+F representative immunoblots of at least three independent experiments are shown. * p<0.05 compared to control cells.

TGF-β1 significantly induced transgelin promoter activity in a dose dependent manner (Figure 3A). The maximum increase of luciferase activity was a 4.6-fold increase with 10 ng/ml TGF-β1 (p<0.05) (Figure 3A). At the transcriptional-level, TGF-β1 also increased transgelin mRNA levels in a dose-dependent manner (Figure 3B). A maximum increase of transgelin mRNA by TGF-β1 was observed at a concentration of 5 ng/ml (10.5-fold increase) after 12 h (p<0.05). At the translation-level, TGF-β1 increased transgelin protein expression in A549 cells in a dose-dependent manner with a maximum increase using 10 ng/ml TGF-β1 after 24 h (Figure 3C). Caffeine was able to inhibit transgelin promoter activity in a dose dependent manner (Figure 3D). At a concentration of 10 mM, caffeine reduced transgelin promoter activity by 71±7% compared to untreated cells (p<0.05) (Figure 3D). At the transcriptional- and translational-level we found a dose- and time-dependent reduction of transgelin mRNA by 85±9% after 12 h (Figure 3E) and a maximal decrease of protein expression after 72 h (Figure 3F) using 10 mM caffeine.

In summary, these data showed that TGF-β1 induced and caffeine inhibited transgelin expression in lung epithelial cells.

### Effect of caffeine and rolipram on TGF-β1 induced transgelin expression in lung epithelial cells

Next, we studied the effect of caffeine and rolipram on TGF-β1 induced up-regulation of transgelin. To evaluate potential effects, promoter-analysis, PCR, and immunoblotting was performed in lung epithelial cells.

**Figure 4:**
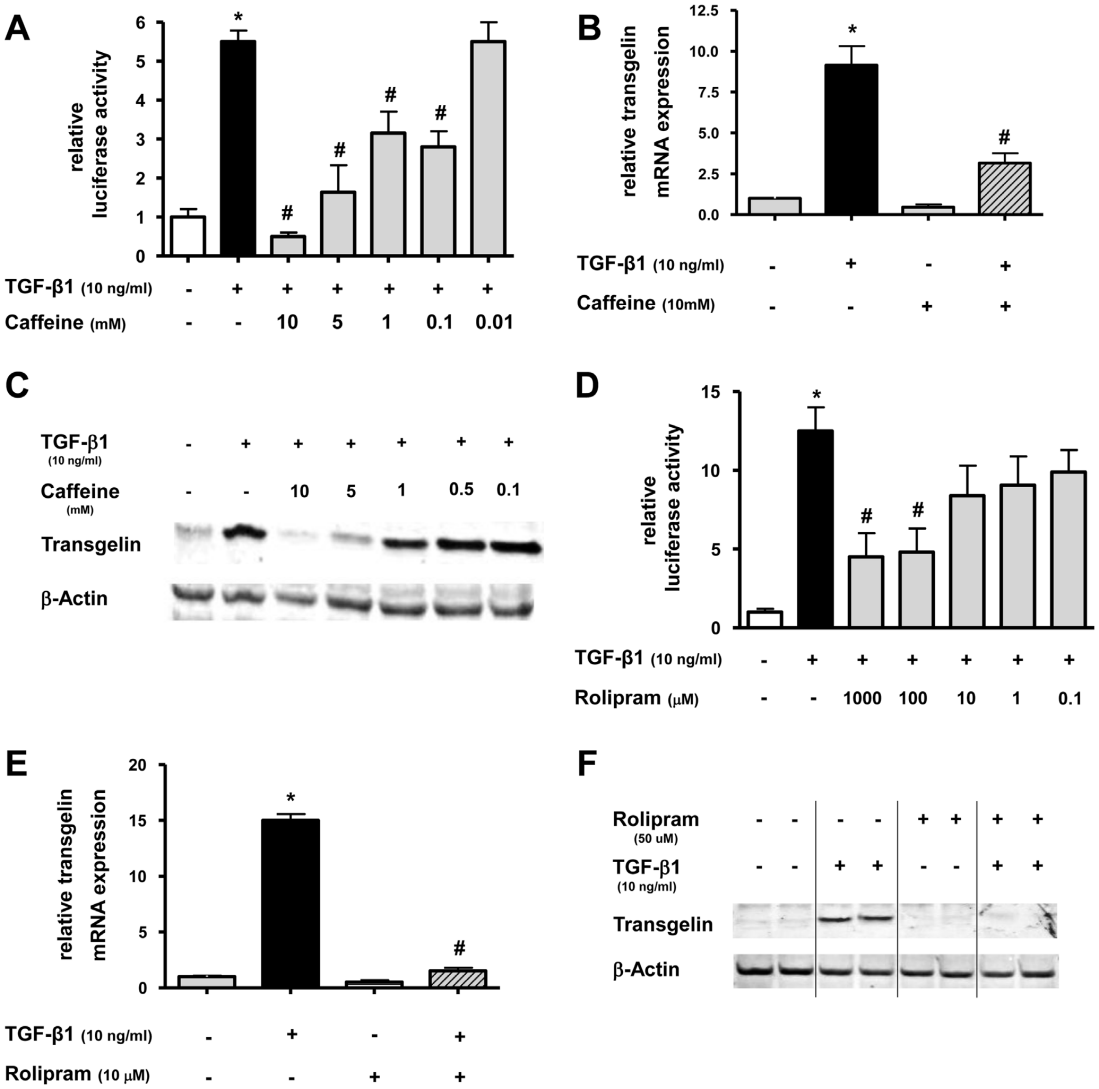
Caffeine and rolipram inhibit TGF-β1 induced transgelin expression in lung epithelial cells. **A+D**: The transgelin promoter luciferase construct was transiently transfected into A549 cells. Cells were then treated with TGF-β1 (10 ng/ml) and/or different concentrations of caffeine (A) or rolipram (D). Firefly luciferase activity was normalized to the activity of Renilla luciferase under control of the thymidine kinase promoter. Relative luciferase activity compared to controls is shown. * p<0.05 compared to control cells, # p<0.05 compared to TGF-β1 treated cells. **B+C/E+F**: A549 cells were incubated with TGF-β1 with or without different concentrations of caffeine or rolipram. Realtime PCR of transgelin mRNA was performed after 12 h (B+E) and Western blot analysis after 72 h (C+F). Relative mRNA levels of transgelin were calculated by normalizing signals to detected GAPDH mRNA (B+E) and compared to untreated cells. Means±SD of at least n = 3 independent experiments are shown. In C+F, representative immunoblots for caffeine (C; triplicates) or rolipram (F; duplicates) against transgelin and β-actin are shown. * p<0.05 compared to control cells, # p<0.05 compared to cells treated with TGF-β1.

For promoter analysis, A549 cells were transfected with a luciferase-reporter construct containing the promoter of the human transgelin gene and treated with TGF-β1 with or without different concentrations of caffeine (Figure 4A) or rolipram (Figure 4D). In the presence of caffeine, TGF-β1-induced stimulation of transgelin promoter activity could be reduced in a dose dependent manner (p<0.05) (Figure 4A). The same was also true for rolipram (Figure 4D) (p<0.05). At 10 mM, caffeine was able to completely antagonize TGF-β1-mediated transgelin expression (p<0.05) (Figure 4A). When rolipram was used at 1 mM, TGF-β1-induced Smad activity was reduced by 64±12% (p<0.05) (Figure 4D). For analysis of mRNA and protein expression, A549 cells were treated with TGF-β1 and with or without caffeine or rolipram. Transgelin mRNA and protein expression were measured 12 or 72 hours later, respectively. Caffeine and rolipram diminished TGF-β1-mediated up-regulation of transgelin mRNA by 63±7% (p<0.05) (Figure 4B) and 90±4% (p<0.05) (Figure 4E), respectively. The inhibitory effect of caffeine and rolipram on TGF-β1 mediated up-regulation of transgelin could also be detected on the protein level by Western blot for caffeine in a dose dependent manner (Figure 4C) and for rolipram (Figure 4F).

Altogether, these data confirmed that both caffeine and rolipram are able to inhibit TGF-β1-mediated up-regulation of transgelin on the promoter-, mRNA-, and protein-level in lung epithelial cells.

### Transgelin silencing with shRNA in lung epithelial cells

**Figure 5:**
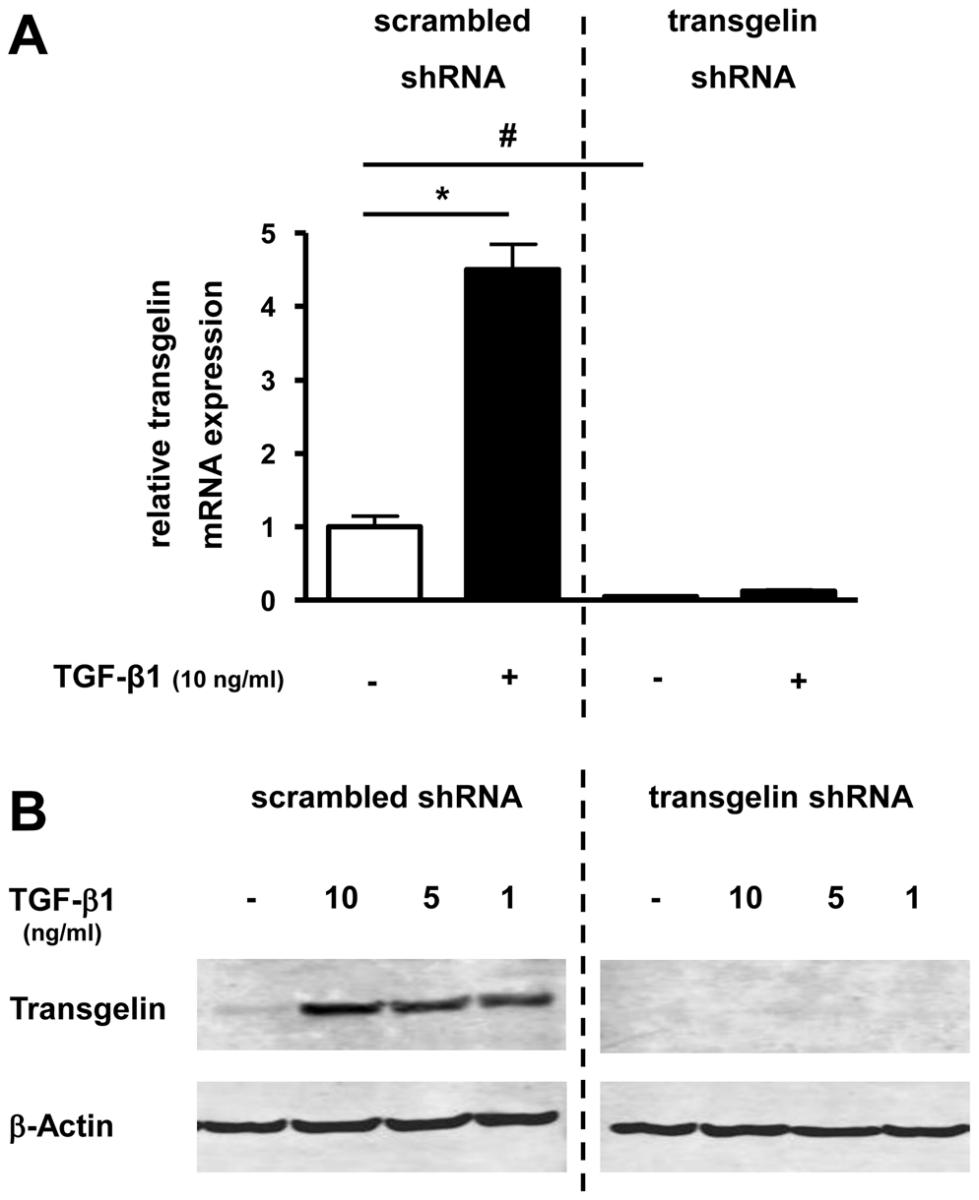
Effect of transgelin-specific shRNA on basal and TGF-β1 mediated induction of transgelin mRNA- and protein-expression in lung epithelial cells. Transgelin was down-regulated by transgelin-specific shRNA (right side; “transgelin shRNA”) compared with control cells, which were expressing non-specific shRNA (left side; “scrambled shRNA”). A549 cells were then cultured in serum reduced medium (0.2% FCS) for 24 h before treatment with TGF-β1. Total RNA and protein was isolated after incubation with TGF-β1 in different concentrations. Transgelin mRNA and protein levels were detected by real time-PCR (after 24 h) (A) or by Western blot analysis (after 48 h) (B), respectively. Means±SD of at least n = 3 independent experiments are shown. In B, a representative immunoblot of at least three independent experiments is shown. * p<0.05 compared to control cells, #<0.05 compared to “scrambled shRNA”.

To study the functional role of reduced transgelin expression in lung epithelial cells, we generated lentiviral vectors mediating expression of transgelin-specific shRNA. Transduction of A549 cells with those lentiviral vectors resulted in a significant decrease of basal transgelin mRNA levels (Figure 5A) in comparison to corresponding scrambled controls. The induction of transgelin mRNA expression after TGF-β1 treatment described above was also inhibited by the transgelin-specific shRNA (Figure 5A). A TGF-β1-induced up-regulation of transgelin on the protein level could also be prevented by transgelin-specific shRNA in comparison to cells expressing non-specific scrambled shRNA (Figure 5B).

These results demonstrated that basal and TGF-β1-induced production of transgelin in lung epithelial cells could be significantly abolished by transgelin-specific shRNA.

### Effect of transgelin gene silencing on Smad signalling in lung epithelial cells

**Figure 6:**
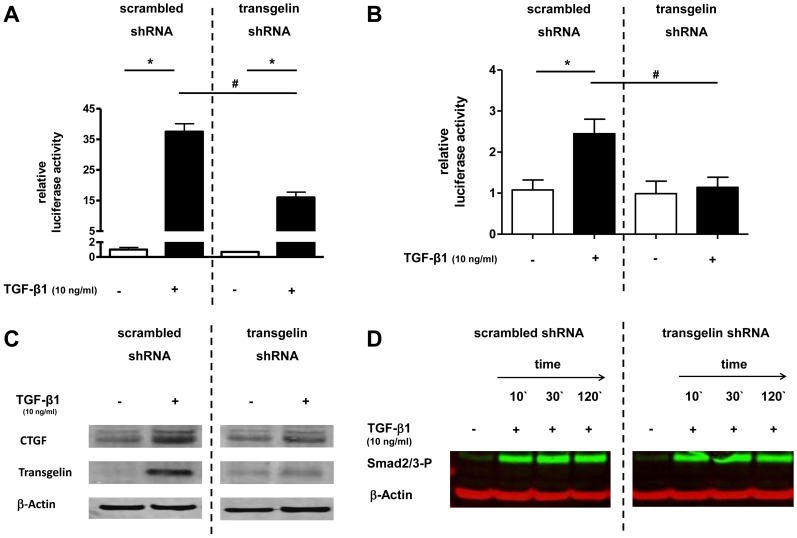
Effect of transgelin silencing on Smad signalling in lung epithelial cells. Transgelin was down-regulated by transgelin-specific shRNA (right side;”transgelin shRNA”) and compared with control cells, which were expressing non-specific shRNA (left side; “control shRNA”). **A+B**. After down-regulation of transgelin by specific shRNA, the TGF-β1–sensitive (CAGA)_12_-luciferase construct (A) or the p3TP-luciferase construct (B) were transiently transfected into A549 cells. Cells were then treated with or without TGF-β1 (10 ng/ml). Firefly luciferase activity was normalized to the activity of Renilla luciferase under control of the thymidine kinase promoter. * p<0.05 compared to control cells, # p<0.05 compared to “control shRNA”. **C**. Cells were incubated with TGF-β1 (10 ng/ml) and 48 h later Western blot analysis of CTGF, transgelin, and β-actin was performed. A representative immunoblot of at least three independent experiments is shown. **D**. Phosphorylation of endogenous Smad2/3 protein. A549 cells were stimulated with TGF-β1 (10 ng/ml) for 10, 30, or 120 minutes. Smad2/3 phosphorylation was detected by immunoblotting with anti-phospho-Smad2/3 antibodies. A representative immunoblot of 3 independent experiments is shown.

To study the possible effect of transgelin on Smad-signalling in lung epithelial cells, the TGF-β1–sensitive (CAGA)_12_- and the p3TP-luciferase construct were transfected into A549 cells in which transgelin expression was knocked down by transgelin-specific shRNA. After TGF-β1 treatment a reduction of TGF-β1-induced luciferase activity to 40% (Figure 6A) or 16% (Figure 6B) was observed in cells expressing transgelin-specific shRNA compared with control cells (p<0.05). The inhibitory effect of transgelin knockdown on TGF-β1 induced CTGF expression could also be confirmed on the protein level by Western blot (Figure 6C). To study the possible influence of transgelin on Smad2/3 protein phosphorylation a western-blot against Smad2/3-P was performed (Figure 6D). No difference in phosphorylation of Smad2/3 could be found between cells with transgelin knock-down and control cells.

## Discussion

In this study, we describe inhibitory effects of caffeine and rolipram on Smad signalling and the expression of TGF-β-regulated genes involved in airway remodelling. Beyond their anti-inflammatory effects, an understanding of these mechanisms might help to explain protective effects of caffeine and rolipram in airway remodelling processes during chronic lung diseases like BPD.

In the neonatal intensive care, methylxanthines including caffeine and theophylline are widely used for the treatment of apnea of prematurity and weaning from the ventilator [23]. Furthermore, reduction of the incidence of BPD in premature infants has been attributed to the use of methylxanthines [23]. In this context, also anti-inflammatory properties of caffeine have been proposed [38]–[40]. Another recently described pharmacological common target for caffeine and glucocorticoids in the lung is the surfactant system [35], [41]. However, until now only a few studies have assessed direct effects of caffeine and PDE-4 inhibitors on molecular mechanisms involved in remodelling processes. Theophylline suppressed TGF-β-induced type I collagen and alpha smooth muscle actin (α-SMA) expression in lung fibroblasts [42]. The anti-fibrotic effects of theophylline are at least partly mediated through the cAMP-PKA pathway [42]. In addition, caffeine and its metabolites suppressed TGF-β-dependent and -independent CTGF expression in the liver via a mechanism that involves reduction of the steady state concentration of total Smad2 protein, decreased phosphorylation of Smad3, and up-regulation of the nuclear receptor peroxisome proliferator-activated receptor (PPAR) γ [43]. Caffeine was able to enforce proteasomal Smad2 degradation by enhancing the activity of Smurf2, a member of the family of E3 ubiquitin ligases with the consequence that Smad2 is increasingly bound to ubiquitin and proteasomally degraded [43]. Our results confirmed that caffeine can also down-regulate TGF-β1 induced CTGF expression in lung epithelial cells. Beside CTGF, transgelin could be identified as another new caffeine-regulated gene involved in airway remodelling processes after lung injury.

Transgelin, also called SM22α, was first described as a 22 kDa protein of unknown function in smooth muscles [44]. Its expression has been detected in several types of epithelial cells [45] including the lung epithelial cells line A549 [18]. A549 cells have been used in several other studies to analyze different aspects of airway remodelling, especially in defining roles of PDE-4 inhibitors [46]–[48]. It is known that A549 cells express high levels of PDE-4, lower levels of PDE-1 and PDE-3, and have a minor PDE-5 activity [48]. Intracellular transgelin is localized in the cytoskeleton apparatus and binds to actin filament bundles [49]. Upon tissue injuries, transgelin is increasingly expressed in lung epithelial cells and may contribute to epithelial mesenchymal transition (EMT) in lung fibrosis [18]. EMT, in which fully differentiated lung epithelial cells change to fibroblasts and myofibroblasts, plays an important role not only in lung development but also in wound healing and lung fibrosis [46], [50]. TGF-β1 can induce transgelin expression and the Smad3/4 complex is involved in the immediate response of the transgelin promoter to TGF-β1 induction [13]. We confirmed the formerly described TGF-β1-induced transgelin expression in lung epithelial cells by Ye et al. [18]. As a new aspect, we showed that basal expression and TGF-β1 induced up-regulation of transgelin could be blocked by caffeine and rolipram.

To analyze the function of transgelin in A549 cells, we modified transgelin expression by transgelin mRNA knockdown using RNA interference. We found that knockdown of transgelin using specific shRNA reduced TGF-β1 induced Smad-signalling and CTGF expression by more than 50%. Similar, Yu et al. described that TGF-β induced migration of alveolar epithelial type II cells could be reduced by transgelin knockdown [18]. It was hypothesized before that there could be a possible association between the TGF-β signalling pathway and the actin cytoskeleton, especially with transgelin [22]. A possible connection between the proposed reduction of transgelin expression and an impaired Smad signalling could be confirmed in our experiments. Our results demonstrated that transgelin expression can influence TGF-β induced Smad-specific gene transactivation, which we have shown in two different Smad dependent luciferase assay and by the regulation of the TGF-β regulated gene CTGF. However, TGF-β mediated Smad2/3 phosphorylation remained unaffected in transgelin knock down cells. A possible explanation of this results could be that the cytoskeleton protein transgelin could influence nuclear translocation of the Smad2/3P complex in the nucleus, DNA binding of the Smad2/3P complex, or the interaction of Smad2/3P with transcriptional co-activators like CBP/p300. Further studies are planned to investigate possible molecular mechanisms involved in interactions between Smad molecules and the cytoskeleton protein transgelin in more detail.

Since the here described effects of caffeine on Smad signalling and CTGF/transgelin expression could only be found at high caffeine but physiological rolipram concentrations, the effect of caffeine on Smad signalling seemed to be mediated by an inhibition of the PDE-4, leading to an increase of intracellular cAMP. The cAMP pathway was described before as a possible regulator of TGF-β-mediated effects [51], [52]. Treatment of human dermal fibroblasts with db-cAMP or forskolin, an established artificial activator of adenylate cyclase and inducer of increased cAMP levels, antagonized inductive effects of TGF-β on the expression of the prototypical TGF-β-responsive genes collagen, CTGF, tissue inhibitor of matrix metalloproteinase-1, and plasminogen activator inhibitor type I (PAI-1)[52]. In our study we could also mimic the effect of caffeine on Smad signalling by rolipram and db-cAMP. In addition, Schiller et al. could show that increased intracellular cAMP prevented TGF-β induced Smad-specific gene transactivation, although TGF-β-mediated Smad phosphorylation and nuclear translocation were not unaffected [52]. However, increased cAMP levels are able to inhibit TGF-β induced interaction of Smad3 with its transcriptional co-activator cAMP-response element-binding protein (CREB)-binding protein (CBP)/p300 [51], [52]. These results suggest that suppression of TGF-β/Smad signalling and resulting gene transactivation by cAMP-inducing agents like caffeine or rolipram could occur via PKA-dependent, CREB mediated disruption of the SMAD-CBP/p300 complex [51], [52].

A limitation of this study is that the effect of caffeine could only be observed at high concentrations. In contrast, treatment of premature infants with caffeine can result in serum concentrations of 100-500 µM [53]. Although a concentration dependent effect of caffeine on liver and lung tissue injury could be described being more protective at higher concentrations and exacerbating at lower concentrations [54], [55], concentrations used in this study had to be high (5–10 mM) to be effective and are thus therapeutically impracticable. Therefore as a second pharmacological substance, the respective capacities of rolipram, a more exclusive PDE4-inhibitor of the first generation, were investigated. Our results showed that an inhibition of Smad-signalling and reduction of CTGF/transgelin expression by caffeine, could also be detected for rolipram. For the latter however, effective concentrations were in a more physiological range and not toxic in comparison to those of caffeine.

As described before by other groups, TGF-β1 is able to induce Smad3 phosphorylation in A549 cells [46]. However, Smad3 phosphorylation could not be blocked by treatment with rolipram at low concentrations (0.1–1 µM) as described in our study [46]. In our test system, we also could not detect an effect of rolipram on Smad signalling at these low concentrations. However, another study showed that PDE-4 inhibitors are potent antagonists of TGF-β signalling and therefore this is in accordance with our results. Here, the second generation PDE-4 inhibitor roflumilast antagonized the induction of CTGF, collagen I, and fibronectin by TGF-β in human airway smooth muscle cells [56]. This effect was different from other applied medications during treatment of chronic lung diseases like corticosteroids or long-acting β2-agonists because those drugs could not prevent the accumulation of TGF-β–induced proteins in the lung [56]. Quite contrary, corticosteroids alone induced expression of CTGF, collagen I, and fibronectin [56]. In addition, rolipram could inhibit epithelial-mesenchymal transition (EMT) in a Smad independent manner in A549 cells [46]. Another aspect is that PDE-4 inhibitors were also effective in inhibiting the secretion of pro-matrix metalloproteinases (pro-MMP-1 and pro-MMP-2) induced by TNF-α [57]. Tissue destruction, which occur secondary to airway remodelling inflammation, has been linked to overexpression of matrix metalloproteinases (MMPs). Our own group described an additive effect of rolipram on steroid induced SP-B expression, which might explain a possible protective effect of PDE-4 inhibitors on lung homeostasis [35].

These in vitro observations and the here described inhibitory effects of rolipram on Smad signalling and CTGF/transgelin expression in lung epithelial cells underline that PDE-4 inhibitors may offer a novel therapeutic strategy for respiratory diseases and could possibly also provide a therapeutic option for very preterm infants with BPD. The effect of different PDE-4 inhibitors (e.g. rolipram, piclamilast, cilomilast) was also investigated in different animal models in which BPD was induced by hyperoxia [27]–[31] or chorioamnionitis [58]. In these studies, PDE-4 inhibitors either protected against BPD or reversed the aberrant remodelling of the alveolar architecture in response to injury. In addition, PDE-4 inhibition was shown to have anti-inflammatory properties, to attenuate pulmonary fibrin deposition and vascular alveolar leakage, and to prolong survival in hyperoxia-induced neonatal lung injury [30]. These findings are also in agreement with observations that PDE-4 inhibition reduced inflammatory response in various other animal models of acute pulmonary inflammation, including lipopolysaccharide (LPS) and antigen-induced lung injury [59], [60].

## Conclusions

It is tempting to speculate that apart from anti-inflammatory effects, caffeine and rolipram may directly antagonize aberrant Smad signalling which in turn improves pathological changes in alveolar architecture observed in BPD or other chronic inflammatory lung diseases. Selective PDE-4 inhibitors like rolipram may have the potential to improve beneficial effects of theophylline and caffeine thereby reducing adverse effects, although existing inhibitors appear to be limited by similar problems [61]. To overcome these pitfalls, administration of more subtype-selective PDE-4 inhibitors by the inhalation route may be the ideal approach to decrease those side effects and to similarly enhance anti-inflammatory and protective properties on airway remodelling processes [62], [63]. In this context, topical administration of PDE-4B selective inhibitors may probably have a superior therapeutic ratio in the future [26], [64].
